# Autonomous Powered Ankle Exoskeleton Improves Foot Clearance and Knee Hyperextension After Stroke: A Case Study

**DOI:** 10.1109/tmrb.2024.3503893

**Published:** 2024-11-21

**Authors:** Kai Pruyn, Rosemarie Murray, Lukas Gabert, Tommaso Lenzi

**Affiliations:** Department of Mechanical Engineering and the Utah Robotics Center, The University of Utah, Salt Lake City, UT 84112 USA; Rocky Mountain Center for Occupational and Environmental Health, Salt Lake City, UT 84111 USA; Department of Mechanical Engineering and the Utah Robotics Center, The University of Utah, Salt Lake City, UT 84112 USA.; Department of Mechanical Engineering and the Utah Robotics Center, The University of Utah, Salt Lake City, UT 84112 USA; Rocky Mountain Center for Occupational and Environmental Health, Salt Lake City, UT 84111 USA; Department of Mechanical Engineering, the Utah Robotics Center, and the Department of Biomedical Engineering, The University of Utah, Salt Lake City, UT 84112 USA; Rocky Mountain Center for Occupational and Environmental Health, Salt Lake City, UT 84111 USA.

**Keywords:** Rehabilitation robotics, wearable robotics, prosthetics and exoskeletons

## Abstract

Hemiparetic gait is often characterized by ankle weakness, resulting in decreased propulsion and clearance, as well as knee hyperextension. These gait deviations reduce speed and efficiency while increasing the risk of falls and osteoarthritis. Powered ankle exoskeletons have the potential to address these issues. However, only a handful of studies have investigated their effects on hemiparetic gait. The results are often inconsistent, and the biomechanical analysis rarely includes the knee or hip joint or a direct clearance measure. In this case study, we assess the ankle, knee, and hip biomechanics with and without a new autonomous powered ankle exoskeleton across different speeds and inclines. Exoskeleton assistance resulted in more normative kinematics at the subject’s self-selected walking speed. The paretic ankle angle at heel strike increased from 10° plantarflexed without the exoskeleton to 0.5° dorsiflexed with the exoskeleton, and the peak plantarflexion angle during swing decreased from 28° without the exoskeleton to 12° with the exoskeleton. Furthermore, stance knee flexion increased from 7° without the exoskeleton to 20° with the exoskeleton. Finally, foot clearance increased with the exoskeleton for all conditions between 3.1 cm and 5.4 cm. This case study highlights new mechanisms for powered ankle exoskeletons to improve hemiparetic gait.

## I. Introduction

STROKE is the leading cause of long-term disability in the U.S. [1], with 80% of stroke survivors experiencing hemiparesis—muscle weakness in one side of the body [2]. Hemiparetic gait is generally asymmetric and inefficient, characterized by foot drop, knee hyperextension, and reduced propulsion [3], [4], [5]. Unfortunately, existing assistive technology cannot fully compensate for hemiparesis. For example, ankle-foot orthoses (AFOs) can stabilize the affected ankle joint and increase toe clearance [6], which is essential for decreasing fall risk. However, they have not been shown to improve knee hyperextension or symmetry in step length or stance time [4], [5], [7]. AFOs have also been shown to prevent normative propulsion and range of motion, most notably on inclines [5]. These limitations have encouraged the development of new assistive technologies.

Powered exoskeletons have the potential to address the needs of individuals with hemiparesis [8]. Specifically, ankle exoskeletons can help the hemiparetic leg by providing plantarflexion and dorsiflexion assistance. Studies with healthy adults have shown that ankle exoskeleton assistance can improve the metabolic cost of walking [9], [10]. However, few studies have examined the effects of ankle exoskeletons on hemiparetic gait.

Ankle exoskeletons are often designed to provide only plantarflexion assistance to improve paretic ankle propulsion and the metabolic cost of walking [11], [12], [13]. Multiple studies have shown that plantarflexion assistance increases propulsion in subjects with hemiparesis [11], [12], [14]. However, only one study has shown improved metabolic cost of walking via assistance from a tethered soft ankle exosuit [15]. Untethered soft ankle exosuits have utilized plantarflexion assistance to improve the maximum and self-selected walking speeds of hemiparetic subjects, but without a decrease in metabolic cost [16], which may be due to the added weight or reduced power. Although paretic ankle power has been shown to improve across different walking speeds [13], the effect of plantarflexion assistance may be even more critical for propulsion walking uphill, which has yet to be examined in individuals with hemiparesis. Previous research has not fully restored ankle plantarflexion propulsion or power back to normative levels, nor has it focused on restoring normative kinematics, for example, ankle joint range of motion. Thus, plantarflexion assistance has the potential to increase paretic ankle propulsion, but the effectiveness may depend on the specific device and controller.

Dorsiflexion assistance from powered ankle exoskeletons has been shown to increase the toe clearance of hemiparetic subjects [8]. However, in the two studies that specifically measured toe clearance, it increased by an average of 2 cm [8] or only a few millimeters [14], which may have limited benefit in the real world. Rather than explicitly measuring toe clearance, it is more common to estimate clearance from the ankle dorsiflexion angle during swing [11], [15]. However, the relationship between minimum foot clearance and dorsiflexion angle is not well established and likely depends on assistance timing. Thus, dorsiflexion assistance has the potential to increase clearance, but we need to understand the effect of the assistance to maximize improvements.

Biomechanical analyses with ankle exoskeletons rarely include the knee and hip joints, even though knee hyperextension is a common compensatory movement used by individuals with hemiparesis. Hyperextension increases the load on the knee and can lead to secondary conditions such as osteoarthritis [17], [18]. To the best of our knowledge, only two studies with hemiparetic subjects included knee biomechanics and showed that ankle exoskeleton assistance did not affect knee kinematics [12], [13]. Thus, the effects of ankle assistance on knee biomechanics require further investigation.

In this case study with one subject with hemiparesis, we investigate the effects of ankle exoskeleton assistance on the ankle, knee, and hip joints. We hypothesize that the autonomous powered ankle exoskeleton presented here can increase foot clearance and propulsion and decrease knee hyperextension by providing plantarflexion assistance during push-off and dorsiflexion assistance during swing. The results of this case study will inform future exoskeleton control development and clinical study design.

## II. Methods

### A. Subject Information

One subject with hemiparesis was recruited for this case study (female, 23 years old, 64 kg, 168 cm, 15 years post-stroke, right side hemiparesis). The subject does not wear an ankle-foot orthosis (AFO) in everyday life and did not wear one for this study. The Institutional Review Board at the University of Utah approved the study protocol. The subject provided informed consent to participate in the study, as well as the use of photos and videos from the experiment.

### B. Experimental Protocol

The experiment was performed in a motion capture lab using a 12-camera Vicon system (Vicon Motion Systems Ltd, Oxford, U.K.) and a fully instrumented split-belt treadmill (Bertec, USA). The subject wore tight-fitting clothing with reflective markers placed on anatomical bony landmarks. We used a modified Plug-in-Gait model [19]. All data were synchronized using a lock sync box (Vicon Motion Systems Ltd, Oxford, U.K.).

We performed a static model calibration and a functional joint calibration, where the subject performed a series of lower-limb joint rotations. The functional calibration locates the centers of rotation for the ankle, knee, and hip joints using the Symmetric Center of Rotation Estimation (SCoRE) and Symmetrical Axis of Rotation Analysis (SARA) [20], [21].

After the calibration trials, we began data acquisition. The subject walked on the treadmill at their self-selected walking speed (1 m/s) for about two minutes. The last minute of the session was recorded. The subject rested for five minutes and donned the ankle exoskeleton, repeating the static and functional joint center calibrations. The subject practiced walking with the exoskeleton for one minute while the experimenter manually set the level of assistance, and then the last minute of the walking session was recorded.

The walking trials were repeated at a fast speed (1.3 m/s) and slow speed (0.7 m/s), as well as at two inclines (5° and 10°) at the subject’s self-selected walking speed (1 m/s). All trials without the exoskeleton were completed before donning the exoskeleton and repeating the trials with exoskeleton assistance. The subject rested between all trials. The subject wore the same shoes with and without the ankle exoskeleton.

### C. Powered Ankle Exoskeleton

**Fig. 1.**
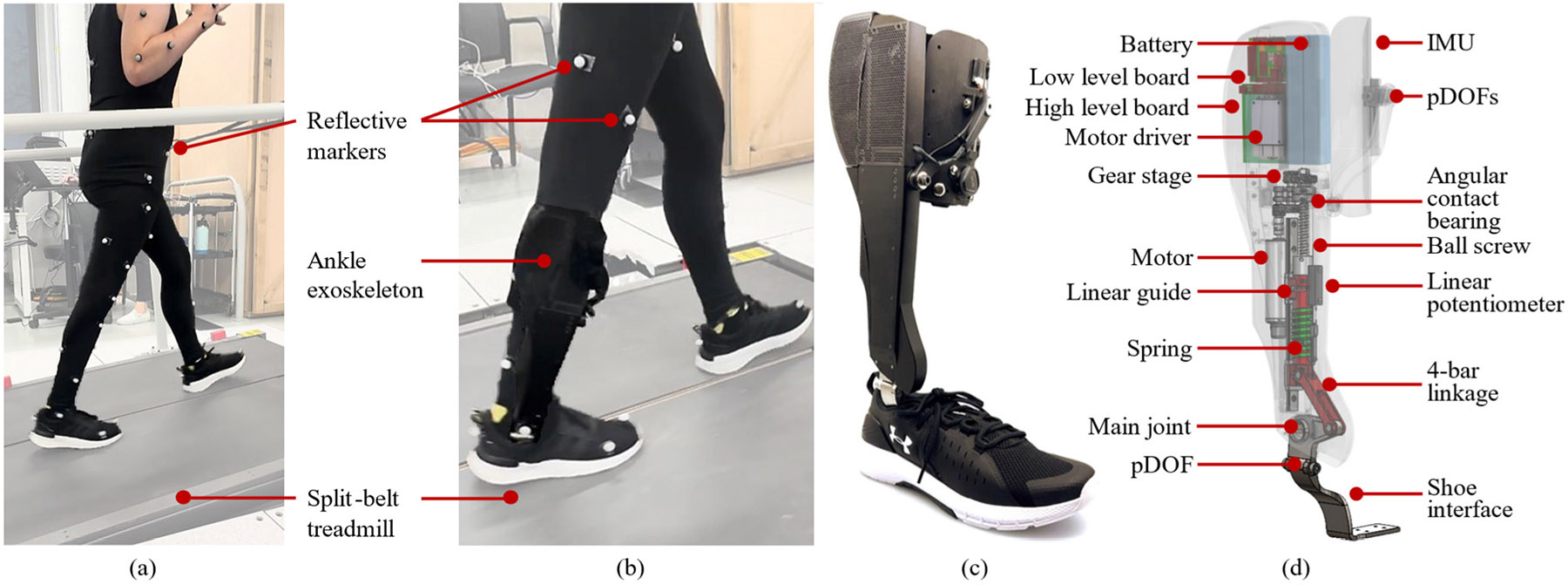
Experimental setup. (a) The participant walking on an inclined treadmill wearing motion capture markers during the experiment. (b) Close-up view of the ankle exoskeleton on the participant. (c) The Utah Ankle Exoskeleton. (d) CAD model of the Utah Ankle Exoskeleton.

To assist the subject’s paretic ankle joint, we used the Utah Ankle Exoskeleton, an autonomous and self-contained powered ankle exoskeleton (Figure 1). The Utah Ankle Exoskeleton features fully integrated series-elastic actuation, batteries, and electronics [22]. The exoskeleton frame connects to the user’s shank through a plastic cuff interface. The distal end of the exoskeleton attaches to a plate that sits under the insole of the user’s shoe. The device can provide up to 40 Nm of torque during level-ground walking. The total weight of the exoskeleton, including the power supply (8S Li-Ion battery), electronics, and interfaces, is 1510 g. The Utah Ankle Exoskeleton uses an embedded microcontroller (PIC32MK0512MCF100, Microchip Technology, USA) to communicate and process sensor data and send low-level commands to the motor control board. An 18-bit off-axis magnetic encoder reads the main joint position (iC-MU DFN16- 5x5, iC-Haus, Germany). Processed orientation data is streamed from an inertial measurement unit (MTi-1, Movella, Netherlands) located on the shank interface. An embedded computer (Raspberry Pi Compute Module 4, Raspberry Pi Foundation, U.K.) communicates over Wi-Fi with an external laptop that can be used to send commands, visualize data, and tune assistance.

### D. Assistive Controller

**Fig. 2.**
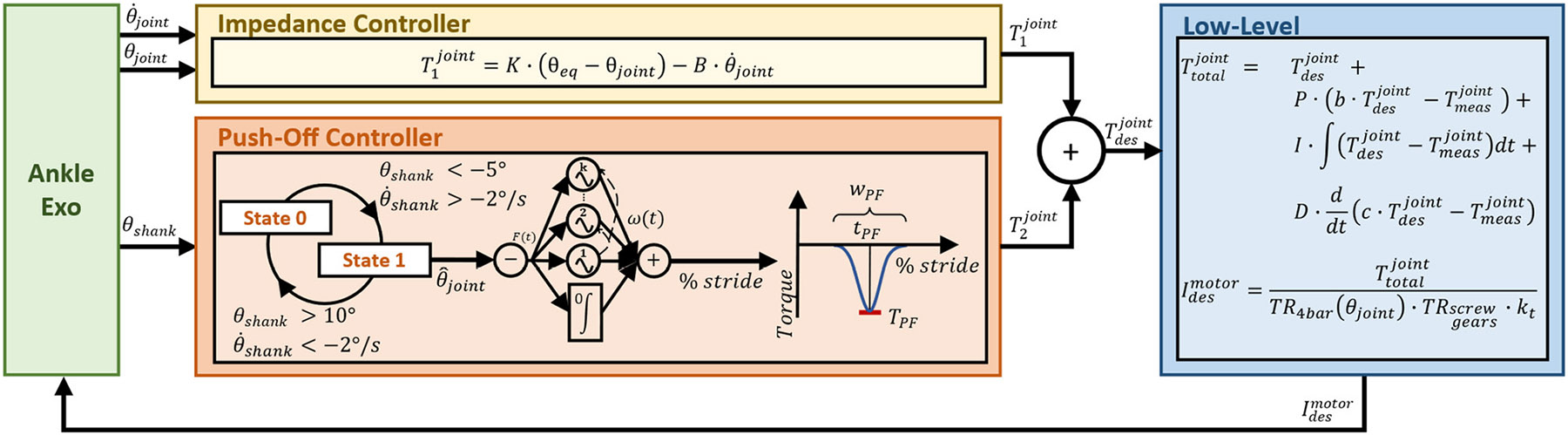
Block diagram of the assistive controller. The impedance controller uses given stiffness and damping values to return the ankle joint to a neutral position. The finite state machine determines the percent of phase used in the adaptive frequency oscillator to provide a Gaussian-shaped curve of plantarflexion assistance during push-off. The torques from each controller are summed to define the desired torque. At the low level, a two-degree-of-freedom closed-loop controller tracks the desired torque and calculates the desired motor current.

The exoskeleton’s embedded control system runs a hybrid torque-impedance controller that provides synchronized dorsiflexion and plantarflexion torque throughout the gait cycle (Figure 2). The impedance controller enforces a neutral equilibrium angle using stiffness and damping values set by the experimenter and fixed throughout the whole gait cycle. The torque controller provides ankle push-off in late stance based on adaptive frequency oscillators [23], [24]. The total desired assistive torque is defined as the sum of the impedance and torque controllers. Both controllers are manually tuned based on feedback from the subject and the experimenter’s experience. At the low level, a two-degree-of-freedom (2-DOF) closed-loop controller tracks the desired torque based on the measured torque estimated by the deformation of the series spring [25].

### E. Data Processing

Experimental data were analyzed with Vicon Nexus 2 (Vicon Motion Systems, Ltd., Oxford, U.K.), Visual 3D (C-Motion, Maryland, USA), and MATLAB (The MathWorks, Inc. Massachusetts, USA). Core processing in Nexus produced 3D trajectories from the raw marker data and calibrated joint positions using SCoRE and SARA. The marker trajectories, force plate analog data, and exoskeleton data were imported into Visual 3D. Kinematics and kinetics were computed using the V3D Composite Pelvis [26]. All inverse dynamic calculations were computed in Visual 3D and imported into MATLAB. As in previous exoskeleton studies [27], we calculated the biological torques by subtracting the assistive torque measured by the exoskeleton from the ankle torque calculated through inverse dynamics [28], [29]. After time normalization, we averaged the processed data across the strides of each trial for both the *No Exo* (gray) and *Exo* (red) conditions. Finally, we quantified the subject’s symmetry using a symmetry index (SI) [30]. The spatiotemporal data from the subject’s non-paretic ($X_{np}$) and paretic ($X_{p}$) sides define interlimb symmetry (1).


(1) $$SI=\frac{X_{np}-X_{p}}{0.5(X_{np}+X_{p})}\cdot 100(\%)$$

A positive SI indicates asymmetry towards the subject’s unaffected side, while a negative SI indicates asymmetry towards the subject’s affected side. Zero indicates perfect symmetry. Minimum foot clearance was determined by the toe position in early- to mid-swing while the knee was flexed, then by the heel position as the knee extended in late swing.

## III. Results

### A. Kinematics

**Fig. 3.**
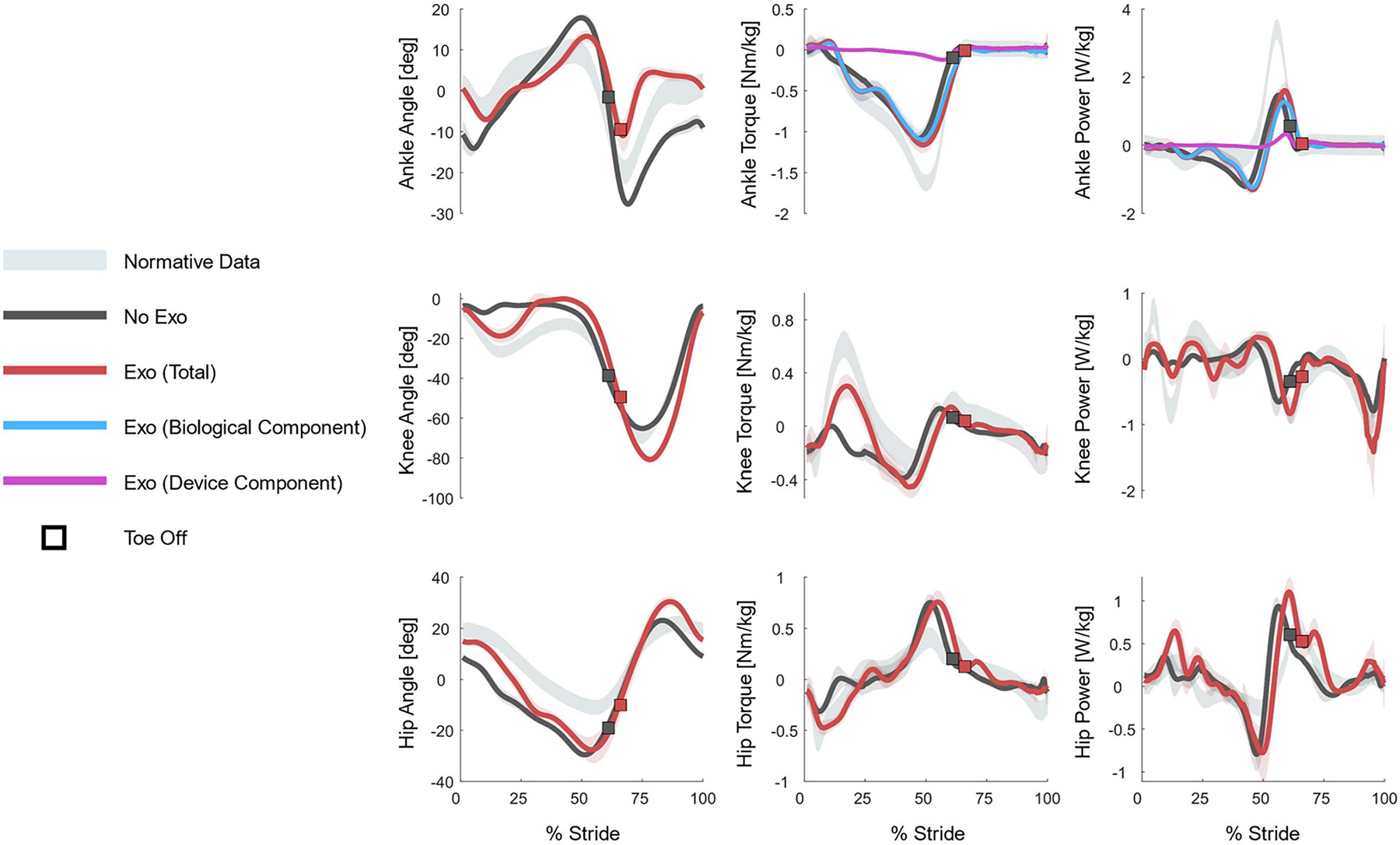
Subject’s affected side kinematics, kinetics, and joint powers during walking at their self-selected speed. Rows, top to bottom: affected ankle, knee, and hip joints. Columns, left to right: lower-limb kinematics, kinetics, and joint powers. The light gray shaded region represents a normative dataset [31]. The *No Exo* condition is shown in dark gray, and the *Exo* condition is shown in red. Toe off for each condition is marked with a square. The ankle torque and power plots include the biological ankle torque or power (blue) and the exoskeleton torque or power (purple). The lines represent the average across strides for the condition, and the colored shaded region is one standard deviation above and below the average. Ankle: positive values represent dorsiflexion. Knee: positive values represent extension. Hip: positive values represent flexion.

Without the exoskeleton, the subject’s gait was characterized by excessive plantarflexion (foot drop) beginning at toe-off and continuing throughout swing and early stance (Figure 3). In contrast, with the exoskeleton, the ankle began dorsiflexing right after toe-off. As a result, the peak plantarflexion angle during swing increased from −28°±1° without the exoskeleton to −12°±3° with the exoskeleton. Accordingly, the affected ankle angle at heel strike increased from −10°±3° without the exoskeleton to 0.5°±0.7° with the exoskeleton, matching the normative data [31].

Without the exoskeleton, the subject had abnormally low knee flexion during early to mid-stance (knee hyperextension). With the exoskeleton, the peak stance knee flexion increased from 7°±1° to 20°±4°, approaching normative data. However, in mid stance, knee extension increased from −2.2°±0.7° without the exoskeleton to 1.8°±0.6° with the exoskeleton. The hip flexion angle at heel strike also increased from 9°±2° without the exoskeleton to 15°±1° with the exoskeleton. With the exoskeleton, the affected hip joint remained flexed for longer during stance rather than immediately extending, as without the exoskeleton.

The kinematic results with the exoskeleton are consistent with trends indicating increased clearance. The mid-swing ankle angle increased from −15°±1° (*No Exo*) to 4°±1° (*Exo*). Furthermore, the knee flexion angle at toe off increased from −43°±3° without the exoskeleton to −52°±4° with the exoskeleton, and the knee velocity at toe off increased by 30% with the exoskeleton. The peak knee flexion during swing also increased, from −65°±1° without the exoskeleton to −80°±2° with the exoskeleton. Finally, the subject stayed in stance longer with the exoskeleton. Specifically, stance lasted 61% of the gait cycle without the exoskeleton and 65% with the exoskeleton.

### B. Kinetics

With the exoskeleton, the affected ankle torque changed most during early stance and push-off. During early stance, the peak biological dorsiflexion torque increased from 0.07±0.06 Nm/kg without the exoskeleton to 0.16±0.11 Nm/kg with the exoskeleton. The exoskeleton provided peak dorsiflexion assistance of 0.06±0.01 Nm/kg at the start of swing. During push-off, the peak of the biological plantarflexion torque decreased by 3.9%, and the peak of the total plantarflexion torque increased by 2.4% with the exoskeleton. The exoskeleton provided a peak plantarflexion torque of −0.14±0.03 Nm/kg, equal to 14% of the total peak plantarflexion torque with the exoskeleton.

The exoskeleton assistance substantially impacted the affected knee torque, including a more normative trajectory due to stance knee flexion. Without the exoskeleton, the knee supplied almost no peak extension torque (0.01±0.03 Nm/kg), whereas with the exoskeleton, it generated 0.29±0.08 Nm/kg. Similarly, the peak of the affected hip extension torque during early stance increased from −0.31±0.05 Nm/kg without the exoskeleton to −0.44±0.05 Nm/kg with the exoskeleton.

### C. Joint Power

The peak biological ankle power decreased by 23% with the exoskeleton, in agreement with the observed reduction in biological plantarflexion torque. However, the peak of the total ankle power was unchanged between conditions. The exoskeleton peak power was 0.35±0.12 W/kg, equal to 28% of the total peak power while wearing the exoskeleton. Finally, there was 21% less negative ankle power with the exoskeleton.

Without the exoskeleton, the affected knee joint power was approximately zero until late stance. In contrast, the knee power profile with the exoskeleton followed a trajectory closer to normative biomechanics, dissipating more energy. For example, the negative knee power peak at the end of swing was −0.81±0.09 W/kg without the exoskeleton, compared to −1.4±0.5 W/kg with the exoskeleton. In contrast, the affected hip joint power increased with the exoskeleton. The positive hip power peaks during early and late stance increased by 61% and 19%, respectively.

### D. Foot Clearance

**Fig. 4.**
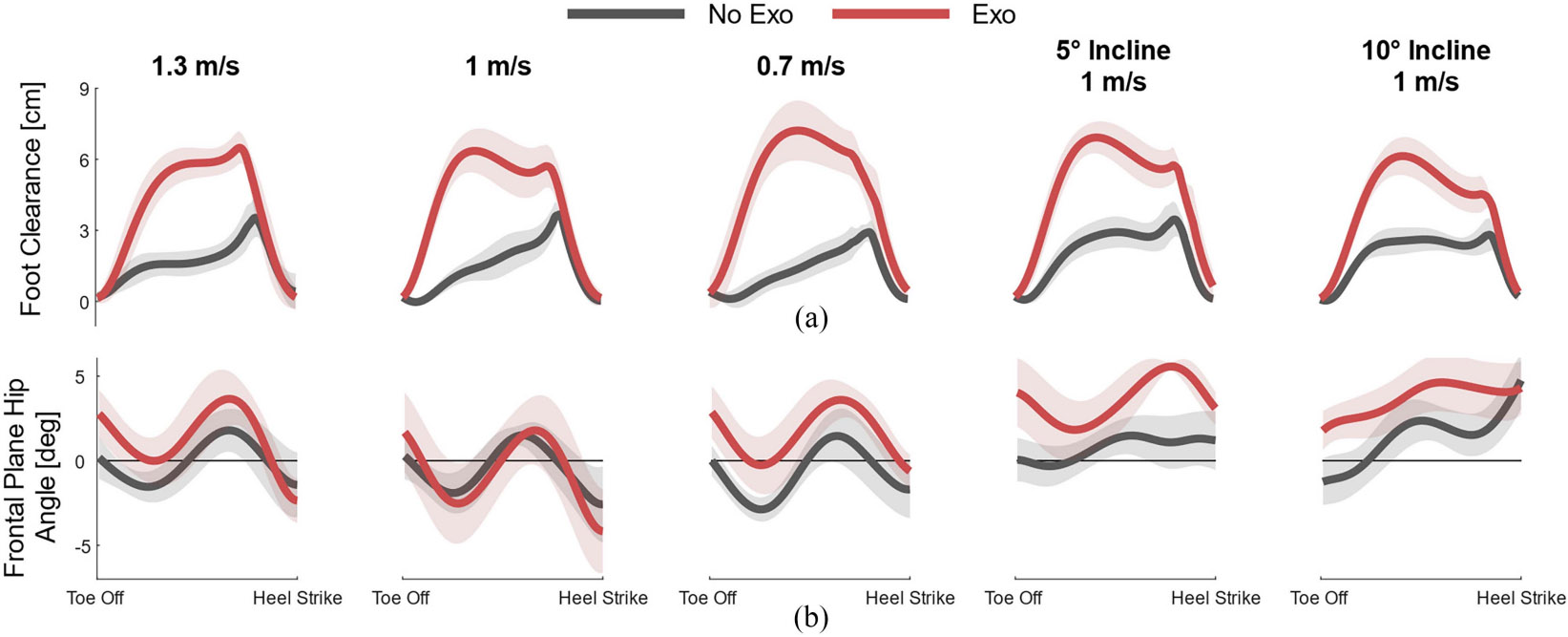
(a) Minimum foot clearance and (b) frontal plane hip trajectories during swing for each walking speed and incline shown as the mean (solid line) and standard deviation (shaded areas) across all strides in the given condition. The *No Exo* condition is shown in dark gray, and the *Exo* condition is shown in red. Adduction is positive, and abduction is negative.

Minimum foot clearance trajectories for walking at all speeds and inclines are shown in Figure 4(a). With the exoskeleton, mid-swing foot clearance increased by between 120% and 303%, equal to multiple centimeters at all speeds and inclines. During fast walking at 1.3 m/s, foot clearance increased by 4.15±0.15 cm. During walking at the subject’s self-selected walking speed (1.0 m/s), foot clearance increased by 3.63±0.10 cm. During slow walking at 0.7 m/s, foot clearance increased by 5.37±0.11 cm. While walking on a 5° incline at 1 m/s, foot clearance increased by 3.83±0.07 cm. Finally, while walking on a 10° incline at 1 m/s, the foot clearance increased by 3.14±0.05 cm.

### E. Frontal Plane Hip Kinematics

Frontal plane hip trajectories during swing for walking at all speeds and inclines are shown in Figure 4(b). During fast walking at 1.3 m/s, abduction at the end of swing increased from −2.4°±0.9° without the exoskeleton to −2.5°±1.2° with the exoskeleton. At 1 m/s, abduction at the end of swing increased from −3.2°±1.3° without the exoskeleton to −4.5°±2.4° with the exoskeleton. During slow and inclined walking, abduction decreased, and adduction increased, with no hip abduction during inclined walking. Mid-swing hip abduction did not increase for all speeds and inclines with the exoskeleton.

### F. Stance Time Symmetry

**Fig. 5.**
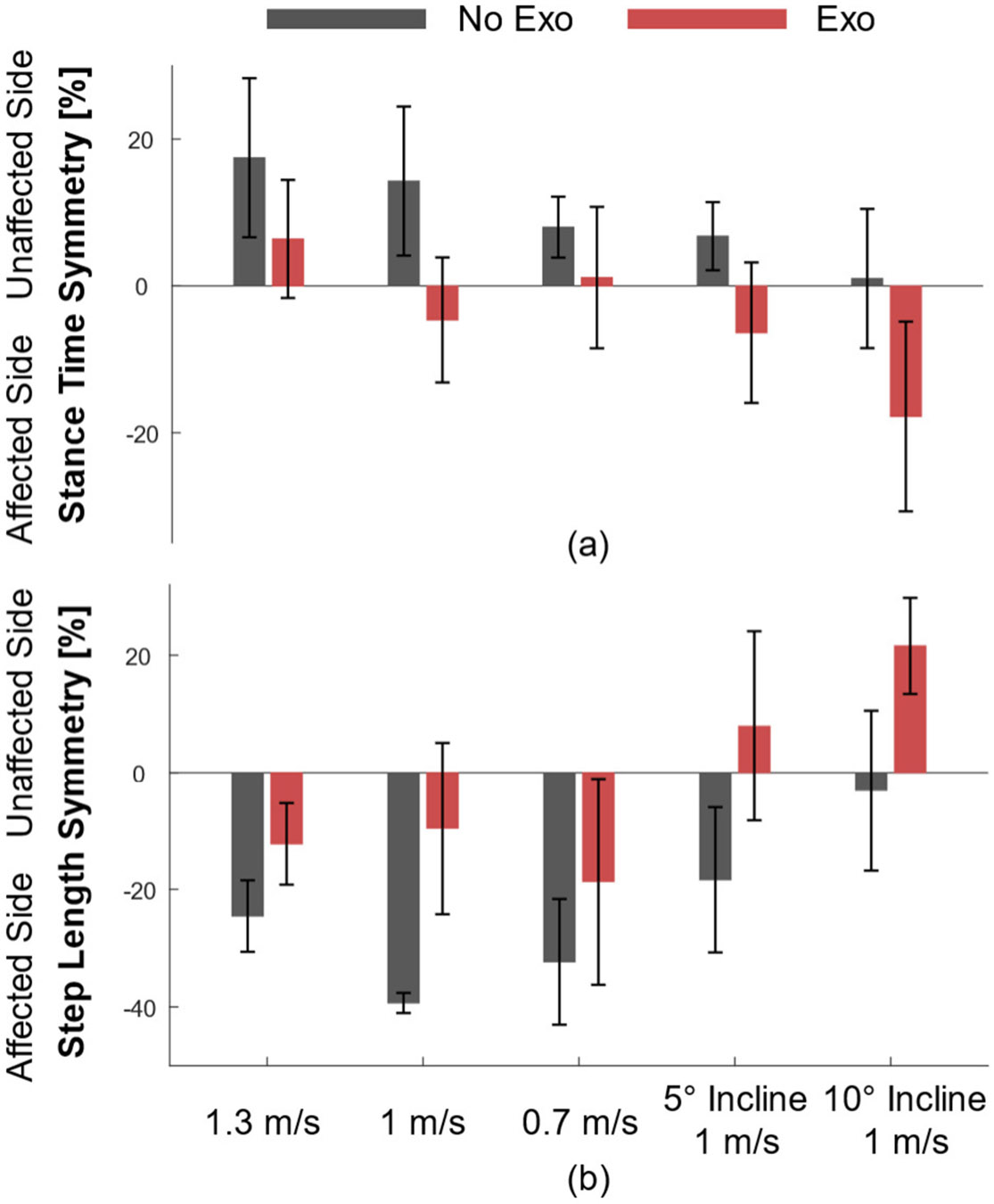
The symmetry index for (a) stance time and (b) step length during each walking speed and incline. The *No Exo* condition is shown in dark gray, and the *Exo* condition is shown in red. (a) Positive values are a longer stance time on the unaffected side and negative values are a longer stance time on the affected side. (b) Positive values are a longer step on the unaffected side, and negative values are a longer step on the affected side.

The stance time symmetry is shown in Figure 5(a). Without the exoskeleton, the subject consistently spends more time in stance on their unaffected side, with improvements in symmetry as the speed decreases or the incline increases. In contrast, the subject spends additional time in stance on the affected side with the exoskeleton to the extent that at 1 m/s at 0°, 5°, and 10° inclines, the subject spends more time on their affected side than their unaffected side. As a result, the absolute stance time symmetry index improved during the *Exo* condition for all walking speeds and inclines, except while walking on the 10° incline at 1 m/s.

### G. Step Length Symmetry

The step length symmetry is shown in Figure 5(b). For all trials, step length without the exoskeleton was longer on the subject’s affected side. With the exoskeleton, the step length symmetry shifts towards the subject’s unaffected side to the extent that at 5° and 10° inclines, the subject takes longer steps onto their unaffected side. Thus, the absolute step length symmetry index improved with the exoskeleton for all tested conditions, except while walking on a 10° incline at 1 m/s.

## IV. Discussion

### A. Significance

Gait after stroke is characterized by deviations from the normative pattern that have a negative impact on mobility. These gait deviations are visible in the ankle, knee, and hip biomechanics of our study participant walking without the exoskeleton (Figure 3). Specifically, without the exoskeleton, we can observe impaired dorsiflexion during swing and early stance, abnormal ankle plantarflexion at heel strike, and knee hyperextension during early stance. Our results show that assistance from a lightweight and compact powered ankle exoskeleton has the potential to reduce these gait deviations by increasing foot clearance and gait symmetry and decreasing knee hyperextension.

The dorsiflexion assistance provided by the exoskeleton enables the subject to have a neutral, normative ankle angle at the end of swing and heel strike. As a result, the subject can roll more naturally over their ankle, maintaining their momentum. As the exoskeleton assistance provides proper support against ankle plantarflexion in early stance, the knee joint can be properly loaded, achieving nearly natural stance knee flexion. This close-to-normative stance knee flexion indicates a more normative weight acceptance phase, which may reduce excessive and undesired loads commonly associated with knee hyperextension in stroke survivors [4]. The increase in knee extension during mid-stance with the exoskeleton likely occurred because the exoskeleton dorsiflexion assistance was too high, making the device too stiff against the subject’s shank. This problem can be addressed by decreasing the stiffness of the exoskeleton dorsiflexion assistance during mid-to-late stance. Although previous studies have demonstrated improved ankle angle at heel strike [12], [14], [15], few include knee biomechanics, none of which show changes in knee position or torque [12], [13]. This study provides a first demonstration that dorsiflexion assistance provided by a powered ankle exoskeleton may have a positive impact on knee hyperextension.

Our results show substantial improvements in foot clearance for all tested speeds (1.3 m/s, 1 m/s, 0.7 m/s) and inclines (5° and 10°). The increase in minimum foot clearance is substantial, ranging from 3.1 cm to 5.4 cm. These improvements are higher than those reported in previous studies, which show foot clearance increasing by only a few millimeters [14] or an average of about 2 cm [8]. Furthermore, the improvements shown here are greater than those from ankle-foot orthoses (AFOs), which have been shown to increase toe clearance by 4 mm [6]. The observed increase in foot clearance seems to be due to both reduced ankle plantarflexion and increased knee flexion in swing. Changes in frontal plane hip angle did not contribute to increased foot clearance. Unsurprisingly, the exoskeleton dorsiflexion assistance directly affected the ankle angle in swing, reducing plantarflexion by almost 20°. More interestingly, our results indicate that plantarflexion assistance may have contributed to the increase in swing knee flexion by assisting swing initiation in late stance. This effect is supported by the 30% increase in knee velocity at toe off. Reduced foot clearance is a marker of increased fall risk among individuals with hemiparesis [32]. This case study suggests that both dorsiflexion and plantarflexion assistance can improve foot clearance, reducing the fall risk of stroke survivors.

Our results show no difference in the total paretic ankle torque and power with and without the exoskeleton. This result is in contrast to previous work showing that the addition of plantarflexion assistance generally increases total paretic ankle torque and power [11], [12], [13]. This difference is likely due to the low level of plantarflexion assistance provided in our study (0.14±0.03 Nm/kg) compared to previous studies (0.22-0.35 Nm/kg [13]). The mechanics of different devices may affect the outcomes, but our results suggest that large changes in range of motion can be achieved from low levels of torque. Further experiments are needed to determine whether increasing the ankle plantarflexion assistance changes the total paretic ankle torque and power during push-off.

Although the total torque did not change, the biological paretic ankle torque and power decreased with the exoskeleton (Figure 3). This result suggests that the plantarflexion assistance allowed the subject’s ankle to relax, which may benefit users with excessive plantar flexor activation. Moreover, this result agrees with a previous study that found plantarflexion assistance decreased paretic soleus activation in individuals with hemiparesis [12]. This case study confirms previous findings that plantarflexion assistance provided by a powered exoskeleton can reduce biological ankle effort.

Our results show that the stance time symmetry index decreased while the step length symmetry index increased for all tested conditions. This result is likely due to a longer stance phase on the affected side, which gave the unaffected side potential for a longer swing phase. In addition, the greater paretic hip extension torque during early stance may have helped propel the unaffected foot farther during swing. These results indicate that the subject can utilize their affected side more with the exoskeleton. However, increased reliance on exoskeleton assistance does not necessarily improve symmetry. For example, on the 10° incline, both symmetry indexes change sign with the exoskeleton and are higher in absolute value, indicating worse symmetry. This result could indicate the need for speed- and incline-dependent tuning of exoskeleton assistance. Further studies are necessary to determine the relationship between exoskeleton assistance and symmetry.

### B. Limitations

Despite the promising results of this case study, there are important limitations to consider. As in all case studies, these findings may not generalize to a broader population, especially given the wide variability observed in individuals with hemiparesis. Additionally, the exoskeleton assistance may not have been optimal, and increased plantarflexion assistance could have produced greater improvements in total ankle torque and propulsion, similar to previous studies. Moreover, the subject had limited time to adapt to the ankle exoskeleton assistance during each condition, with data recorded immediately after. However, it has been shown that it can take hours of training for subjects with mobility challenges to experience maximum benefits from exoskeleton assistance [33].

Future work will repeat the experimental protocol with more hemiparetic subjects to confirm these results statistically. These experiments should include multiple levels of ankle plantarflexion assistance and more training time for better adaptation to the exoskeleton assistance. Additional research quantifying the clinical significance of these findings is another essential step for exoskeletons to assist individuals with hemiparesis effectively. Future analysis should include a more extensive performance assessment with outcome measures like overground walking speed, muscle effort, and the metabolic cost of walking.

## V. Conclusion

This case study shows that plantarflexion and dorsiflexion assistance can improve hemiparetic gait by increasing foot clearance and symmetry in stance time and stride length and decreasing knee hyperextension. Furthermore, our results indicate that the lightweight and compact powered ankle exoskeleton presented in this study effectively assists hemiparetic gait, with the potential to produce meaningful clinical results. This case study highlights new mechanisms for powered ankle exoskeletons to improve hemiparetic gait patterns.

## Supplementary Material

supp1-3503893
